# Discussion of Miller’s Theory at the Last Meeting of the American Dental Society, of Europe

**Published:** 1885-01

**Authors:** 


### Selections.

### Discussion of Miller's Theory at the Last Meeting of the American Dental Society of Europe.

Dr. Miller said: Some Americans who do not investigate for themselves take some authority on micro-organisms, as Pasteur, Tyndall, etc., no matter how old it may be, and base their theoretical views upon this. To study the subject, one must take the more recent German investigators, as Koch, and study for oneself by means of patient cultivation of the organism and by much microscopic observation. One must be sufficiently familiar with the organisms to detect them at sight. I will not go into the subject, as my views have been already sufficiently ventilated, but I would mention, briefly, that the question has arisen as to whether the action of the organism is primary or secondary, it being pretty well proved that dental caries commences in decalcification produced by acid; but whether these organisms secreted the acids, or merely followed where they had preceded, was the question. Let me illustrate. Suppose we have a cavity in a molar, as in sketch. The carious portion is removed by spoon excavators, sliced in the freezing microtone, stained in fushine, cleared by essential oil, and mounted in balsam. These specimens show that the organisms travel only in the radial line with the pulp, or, in other words, in the tubules; that in the corners where the external ends of the tubules are not exposed there we have no organisms, but we do have decalcification, the action of the acid travelling in all directions, while the micro-organisms only follow the line indicated. Undoubtedly the fungi have the power of converting any saccharine matter into lactic acid. Now, if our statements are correct, we should be able to produce artificial caries, and this we can do. I have with me, and will show, at the demonstration to follow, any number of slides where the pathological conditions are identical.

Dr. Du Bonchet: What do you consider the best antiseptic?

Dr. Miller: I would refer to my papers in the *Independent Practitioner* (and *Dental Record*), one of which gives a list of substances tested for their powers in this respect. However, hydrargyrum bi-chloridum is, doubtless, the most potent; but, as it is a powerful poison, it must be used diluted—say 1 per cent.—but it would seem that iodoform fills the requirements as well as it is possible.

Dr. Gregory: Does the decalcified portion you have mentioned, in which there are no micro-organisms, become recalcified?

Dr. Miller: No doubt.

Dr. Terry: Would it not be safe practice to excavate the decalified part and fill with cement?

Dr. Gregory: But the recalcification is certainly better than any artificial substitute.

Dr. Miller: Of course, if in excavating you cut off the tubules from the pulp, you can have no recalcification.

Dr. Bodecker: Recalcification can only occur near the pulp. There must of necessity exist a grade of low inflammation, the same as in all cartilaginous parts, and before recalcification can occur there must be a so-called retrograde metamorphosis—that is, there must be a breaking down of the more complex into the more simple elementary combination, and from the medullary condition advance. I have had very much pleasure in seeing Dr. Miller's beautiful specimens. I have not had the opportunity of making any original research myself in the etiology of dental caries; I shall not, therefore, criticise what has been said.

Dr. Terry: Has Dr. Miller always found the same fungi?

Dr. Miller: No; there are a large number of fungi of different kinds, and they do not like to live together. The particular kind found in human caries can exist without oxygen, and are not instrumental in producing putrefaction.

Dr. Terry: All the varied forms of decay have a cause in organisms, have they not?

Dr. Miller: Yes.

Dr. Terry: Is there, then, one which might be considered normal?

Dr. Miller: That is a very difficult question. The fungi have a separate existence, and are not the result, but the cause, of decay. For example, if you keep beef in an antiseptic condition—that is, keep out the fungi—it will keep indefinitely.

Dr. Terry: All air, then, contains the organisms. Does the air found at different altitudes or in different places contain different amounts of the fungi? Is the air of the Engadine freer than that of Vevay?

Dr. Miller: Yes, undoubtedly the question of temperature, humidity, barometric pressure, etc., are important in determining the amount, but perhaps humidity is the most important. When moist the organisms stick to anything they may come in contact with, but when they become dry they are released and float in the air.

Dr. Terry: The French, in opposition to Dr. Koch's views, water the streets during the cholera epidemic. Are there organisms in the blood?

Dr. Miller: It is difficult to say; in a state of perfect health they probably do not exist, but when any pathological condition is present they may. This is Dr. Koch's view.—*Report in Dental Record for October.*

